# Exergy-energy, sustainability, and emissions assessment of *Guizotia* *abyssinica* (L.) fuel blends with metallic nano additives

**DOI:** 10.1038/s41598-024-53963-8

**Published:** 2024-02-12

**Authors:** M. S. Abishek, Sabindra Kachhap, Upendra Rajak, Tikendra Nath Verma, Nimay Chandra Giri, Kareem M. AboRas, Ali ELrashidi

**Affiliations:** 1 Department of Mechanical Engineering, National Institute of Technology Manipur, Imphal, Manipur 795004 India; 2 Department of Mechanical Engineering, RGM College of Engineering and Technology Nandyal, Nandyala, Andhra Pradesh 518501 India; 3 Department of Mechanical Engineering, Maulana Azad National Institute of Technology Bhopal, Bhopal, MP 462003 India; 4 Department of Electronics and Communication Engineering, Centurion University of Technology and Management, Jatni, Odisha 752050 India; 5 Department of Electrical Power and Machines, Faculty of Engineering, Alexandria University, Alexandria, 21544 Egypt; 6 Electrical Engineering Department, University of Business and Technology, Ar Rawdah, 23435 Jeddah, Saudi Arabia; 7 Engineering Mathematics Department, Faculty of Engineering, Alexandria University, Alexandria, 21544 Egypt

**Keywords:** Exergy analysis, Energy analysis, *Guizotiaabyssinica* (L.) biofuel, Nano additives, Compression ignition engine, Energy storage, Energy science and technology, Renewable energy, Bioenergy, Biofuels

## Abstract

This study extensively examined the impact of aluminium oxide (Al_2_O_3_) and titanium dioxide (TiO_2_) nanoparticles addition in the biodiesel fuel derived from *Guizotia* *abyssinica* (L.) oil. The assessment of fuel blends, which were created by combining nanoparticles and biodiesel was conducted using energy, exergy, and sustainability indices. The highest recorded power output of 2.81 kW was observed for the GAB20A engine operating at 1800 rpm. The experimental results revealed that the GAB20A exhibited the lowest fuel consumption, with a recorded value of 203 g/kWh, when operated at 1600 rpm among all the tested blend fuels. The blend GAB20A exhibited the highest level of energy efficiency at 1600 rpm of 29.5%, as determined by the study. Simultaneously, it was observed that GAB20 exhibited the lowest energy efficiency at 1200 rpm among all the blend fuels at 25%. The emission levels of nitrogen oxides (NOx) and carbon monoxide (CO) were observed to be quite low, although a little rise in carbon dioxide (CO_2_) was detected. For validation of results the artificial neural network (ANN) was used and an average of 1.703% difference in energy efficiency, 2.246% decrease in exergy efficiency, and 1.416% difference in sustainability index was found.

## Introduction

The stringent emission regulations, automobile manufacturers are obligated to regulate and mitigate the release of harmful pollutants originating from diesel engines^1^. The use of bioresources for the production of biofuel is widely regarded as a very promising strategy for achieving cleaner power generation and advancing the global circular bio-economy. The higher cetane rating of biodiesel makes it a viable fuel option for diesel engines that use compression ignition (CI) technology^2^. The biodiesel (fatty acid methyl ester; FAME) profile was evaluated using lipid transesterification, as conducted by Jae-Cheol Lee et al.^3^. The physicochemical properties, and exhaust emissions of several ternary fuel mixes including waste fish oil (WFO) biodiesel, bioethanol, and petro-diesel^4^. The use of biodiesel in a CRDI engine may be achieved by blending, even under varying load conditions. The rise in the proportion of biodiesel has a significant impact on the results of experiments related to the combustion process, efficiency, and pollution^5^. Advancements in nanotechnology have a significant influence on the automobile industry. The ongoing discourse has contributed a new terminology, specifically referred to as “Nano fuels”, to the pre-existing corpus of scholarly works^6^. The nanoparticles that were synthesised utilising the aqueous precipitation approach have been used in many scientific studies^7^. The anti-corrosion properties of ionic fluid are beneficial in managing the corrosion of carbon steel within biodiesel^8^. The nanoparticle is incorporated into biodiesel blends by the use of an ultrasonicator running at a power level of 500 W and a frequency of 20 kHz. The use of this equipment is of significant importance in facilitating the effective amalgamation of nanoparticles with fuel, hence enhancing the overall efficiency of the process. This is achieved by the steady maintenance of a predetermined frequency for a length of 45 min under standard room temperature conditions. The outcome is an enhanced amalgamation of the nanoparticles with the fuel^9^. At elevated temperatures ranging from 700 to 800 °C, there is a notable occurrence of intense micro-explosions. It leads to a substantial augmentation in the rate of evaporation, while using Aluminium (Al) as an additive. The emission parameters of biodiesel exhibited a high degree of similarity to those of diesel fuel. Biodiesel exhibited minimal levels of nitrogen oxide (NOx) emissions, achieving a notable decrease of up to 11.18%^10^. The energy of activation of droplets with greater concentrations of Al nanoparticles (2.5% and 5.0%) exhibits an increase compared to that of droplets consisting only of pure diesel. All nanoparticle additions, exhibited a decrease in NOX emissions in the experimental engine^11^. The maximum recorded reduction in NOX emissions was found to be 16.7% while introducing TiO_2_ nanoparticles at a concentration of 100 ppm. The incorporation of TiO_2_ in diesel fuel led to a noteworthy decrease in brake specific fuel consumption (BSFC) by 22%, Hydrocarbon (HC) emissions by 18%, and Carbon Monoxide (CO) emissions by 25%^12^. The addition of Al_2_O_3_ nanoparticles at a concentration of 40 parts per million (ppm) resulted in a 10.57% increase in the Brake Thermal Efficiency (BTE) and an 11.65% decrease in the Brake Specific Fuel Consumption (BSFC). Furthermore, there was a decrease of 22.84%, in smoke emissions 26.72% in HC emissions and 48.43% in CO emissions. However there was a rise of 11.27%, in NO_X_ emissions^13^. The incorporation of additional into biodiesel has the potential to augment the combustion process via the provision of more oxygen molecules. Furthermore, the issue of elevated viscosity in biodiesel may be alleviated with the use of additive possessing a lower viscosity. Dual fuel engines often encounter a reduction in the availability of intake air, which may be mitigated to some extent by including oxygenated fuel in conjunction with biodiesel^14^. The artificial neural network (ANN) methodology was used to validate the obtained findings^15^^.^

Mixing nanoparticles with biodiesel in controlled environments is crucial for their interaction with engine components. Proper temperature regulation is essential to prevent corrosion. Among the metals, namely aluminum, mild Steel, and copper, aluminum has the highest resistance to diesel, biofuel, or diesel-biofuel fractions^16^. Therefore, the use of aluminum alloy components in conjunction with aluminum-blended gasoline may effectively mitigate the occurrence of galvanic corrosion.

Pure biodiesel in diesel engines has limitations due to high viscosity, low cetane number, cold weather, frequent maintenance, and low brake thermal efficiency. To improve fuel quality, fuel additives have been added over the past decade^17,18^. Alcohol-based additives provide additional oxygen in the combustion chamber, lowering emissions. However, developing a lean mixture reduces the biofuel’s calorific value, leading to reduced engine performance and increased potential for damage when combined with higher auto ignition temperatures and poorer lubricating properties^19,20^. The use of nanoparticles as a constituent in biodiesel fuel enhances the properties of pure biodiesel. Consequently, researchers have undertaken investigations into the feasibility of using nanoparticles as enhancements to augment the attributes of biofuels.

The significance of energy analysis in assessing the efficiency of diesel engines and thermal systems is emphasised in the literature study. However, energy analysis alone is insufficient for a comprehensive understanding of the fuel. The use of exergy analysis provides enhanced precision in obtaining outcomes by taking into account the intrinsic irreversibility’s present within the system. The consideration of improvements in thermal systems is of utmost importance in order to optimise efficiency and sustainability, while also taking into account economic aspects. The oil is derived from the seeds of the botanical species *Guizotia* *abyssinica* (L.). The transesterification process is used for the purpose of converting the oil, leading to the subsequent separation of methyl ester and glycerol. The biodiesel produced is then blended with 80% pure diesel, referred to as GAB20. Nanoparticles composed of aluminium and titanium were separately integrated into GAB20 fuel, along with the surfactants commercially known as Sodium Dodecyl Sulphate (SDS) (CH_3_ (CH_2_)11SO_4_ Na +). The Nano fuel that has been obtained is being subjected to performance and emission tests inside a Multi-fuel variable compression ratio engine. Following this, an investigation was carried out using the empirical data collected to examine the concepts of energy, exergy, and sustainability. The experimental findings were validated by comparing them to the output of the artificial neural network (ANN).

## Methodology and method

### Feedstock and properties

**Figure 1:**
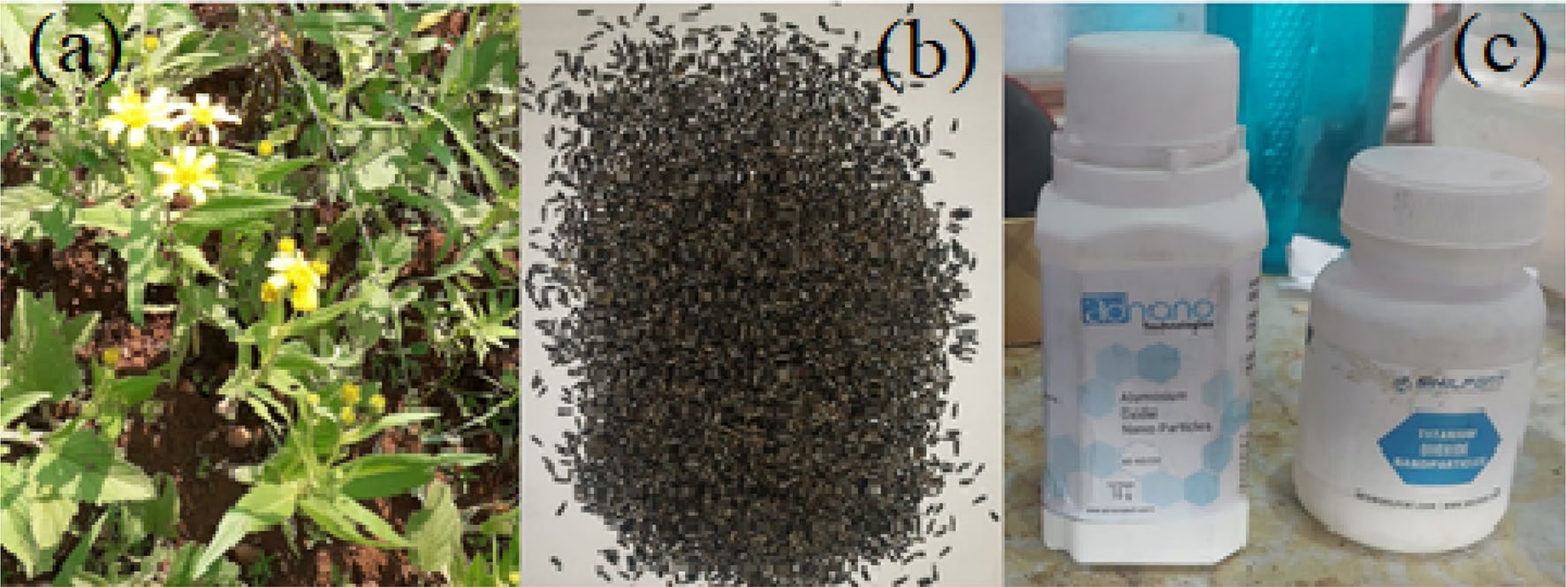
(**a**) *Guizotia* *abyssinica* (L.) plant, (**b**) *Guizotia* *abyssinica* (L.) seed, (**c**) Nanoparticles.

The *Guizotia* *abyssinica* (L.) (GA) seed is primarily planted in certain parts of India and Ethiopia as a rotating crop. The seeds used for the experiment were procured from Patrau, a region located in the Ramgarh district of Jharkhand, India. The aforementioned seed accounts for around 3% of the total oilseed output in India. The maturation period of *Guizotia* *abyssinica* (L.) seed plants ranges from 110 to 120 days, during which they attain a height of around 0.5 to 1.5 m. The cultivation of the crop was observed to be possible in various soil conditions. The cultivation of this seed is often practised in India, namely in regions characterised by acidic soil, poor soil quality, or low fertility mountainous slopes. An average yield of 200–300 kg/ha may still be achieved under suboptimal management circumstances. The seed of *Guizotia* *abyssinica* (L.) is composed of about 40% oil, consisting of 7–8 wt.% stearic and palmitic acids, 75–80 wt.% linoleic acid, and 5–8 wt.% oleic acid^21^. Figure 1a and b depict the *Guizotia* *abyssinica* (L.) plant in its natural habitat and the respective seeds taken from the plant. The Aluminium oxide nanoparticle was purchased from Ad-nano and titanium dioxide nanoparticle was purchased from Shilpent nanoparticle manufactures showed in Fig. 1c. The properties of the nanoparticles is listed in Table 1.

**Table 1:** Nanoparticle properties.

Nanoparticle	Abbreviation	Size	Purity	Thermal conductivity	Density
TiO2	Titanium dioxide	20–30 nm	> 99.5%	8 W/m.K	4.50 g/cm^3^
Al2O3	Aluminium oxide	20–30 nm	> 99.5%	40 W/m.K	3.98 g/cm^3^

### Nano fuel preparation

The oil derived from the seeds undergoes examination to determine the content of free fatty acids (FFA). The acid esterification process is not conducted in instances when the free fatty acid (FFA) content is below 3%. The seed oil derived from *Guizotia* *abyssinica* (L.) undergoes a transesterification process, resulting in the production of *Guizotia* *abyssinica* biodiesel (GAB). The transesterification procedure is conducted for a duration of 60 min, using a 0.1N sodium hydroxide (NaOH) catalyst and maintaining a methanol to oil ratio of 10:1. During the course of the reaction, a consistent rotational velocity of 500 revolutions per minute (rpm) was meticulously maintained at a temperature of 60 °C. The separation of biodiesel and glycerol was accomplished by using a separating funnel. The biodiesel is subjected to a comprehensive rinse procedure and afterwards heated to a specific temperature of 85 °C in order to thoroughly remove any remaining moisture^22^.

Engineering applications encounter challenges when it comes to nanoparticles, such as the costs of acquisition discrepancies, in research findings and a limited theoretical grasp of their properties. The preparation of nanofluids involves either one step or two step methods, they necessitate equipment. The tendency of nanoparticles to sediment into large particles restricts their high surface area, and particle dispersion additives are added to prevent this. However, this method can modify the surface properties of the nanoparticles, resulting in unwanted impurities. Al_2_O_3_ or TiO_2_ nanoparticles have an oxide layer, which can capture larger portions of the particles’ volume, causing energy loss. So, in order to mitigate phase separation and agglomeration, the addition of surfactants is necessary^23^.

The preparation method for nanoparticle dispersion is crucial for improving surface charges and dispersion stability. The zeta potential, a potential created by coating nanoparticles with surfactants, plays a significant role in preventing electrostatic repulsion forces and remuneration for Van der Waals attractions forces. The quantity of surfactants plays a significant role in the constancy and superiority of the dispersion. Ionic surfactants have higher thermal conductivity than non-ionic surfactants, while anionic surfactants can deliver higher values of thermal conductivity. The present investigation utilised commercially accessible analytical grade Sodium Dodecyl Sulphate (SDS) (CH_3_(CH_2_)11SO4 − Na +)^23^. In a blending process, a ratio of 1:2 is used to combine 5 mg of NPs with a surfactant quantity of 10 mg, to achieve better homogeneity of the nanoparticle surface^23^. The temporal variations in absorbance measurements have the potential to provide insights on the stability of the complex formed by the nanoparticle and surfactant^1^. The ultrasonicator was used to effectively combine diesel fuel and nanoparticles, resulting in a uniform dispersion of the mixture. Table 2 provides a comprehensive overview of the factors associated with biofuel and nanoparticle-infused mixtures.

**Table 2:** Properties of fuel used in experiments.

Properties	D100	GAB20	GAB20A	GAB20T	ASTM (D6751)
Density	0.834	0.823	0.848	0.857	0.86 to 0.90
Viscosity (mm^2^/s)	2.91	3.12	2.95	3.01	1.9 to 6
Flash point (0C)	79	81	82	84	Min 51
Calorific value (MJ/kg)	45.02	43.81	44.912	44.196	–

### Engine specification

**Figure 2:**
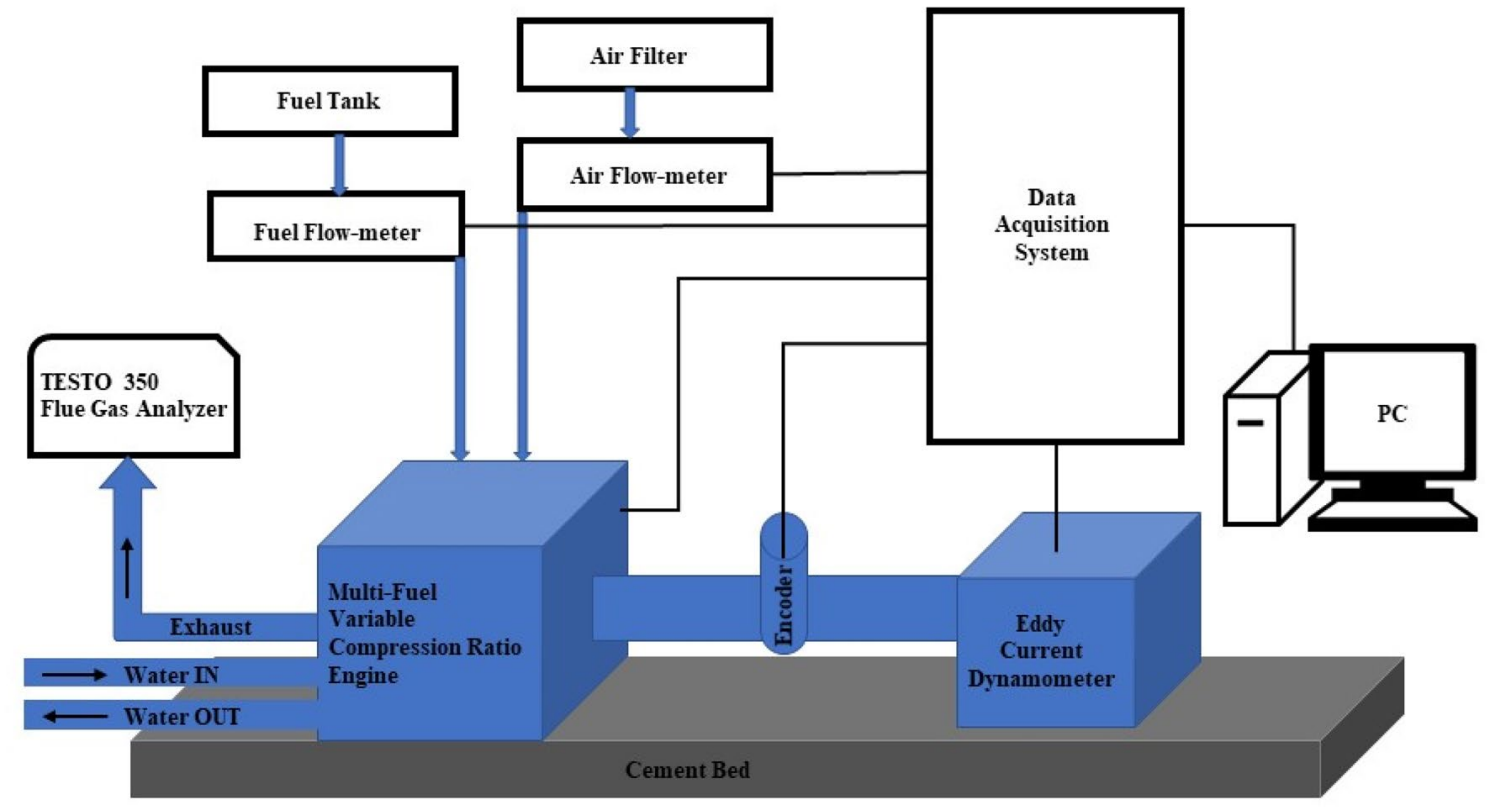
Schematic diagram of experimental setup.

The test engine setup known as “Legion Brothers”, that is used in the Fuel testing procedure. Figure 2 depicts the schematic design of the engine. Table 3 presents the parameters pertaining to the test engine. In accordance with the prescribed engine start-up protocol, a sufficient duration was allocated to allow the engine to attain its ideal operational temperature. Consequently, the system attained a condition of balance. The engine underwent performance testing at several speed levels, namely 1200 rpm, 1400 rpm, 1600 rpm, and 1800 rpm. Prior to initiating the ignition procedure with a novel fuel composition, a designated time interval of 15 min was allocated for the engine to perform operational activities. The objective behind the implementation of this process was to guarantee the thorough burning of any leftover fuel residue from the preceding experiment. Subsequently, meticulous record-keeping was constantly maintained with respect to the measurements of engine speed, fuel consumption, and workload. Furthermore, a thorough evaluation was performed to analyse the braking power and brake-specific fuel consumption (BSFC). The TESTO 350 flue gas analyzer is utilised to measure the concentrations of exhaust emissions, such as particulate matter (PM), carbon monoxide (CO), carbon dioxide (CO_2_), and nitrogen oxides (NO_x_), once the engine has reached a steady operational state. Table 3 shows the engine specification of the engine used to perform the experiments.

**Table 3:** Engine specification.

Parameter	Value	Unit
Make	Legion brothers	–
Engine type	1/4, DI engine	–
Orientation	Vertical	–
Bore × stroke	80 × 110	(mm × mm)
Maximum power	3.7	(KW)
Compression ration	Variable	–
Dynamometer	Eddy current	–
Cooling	Water cooled	–

### Thermodynamic modelling

**Figure 3:**
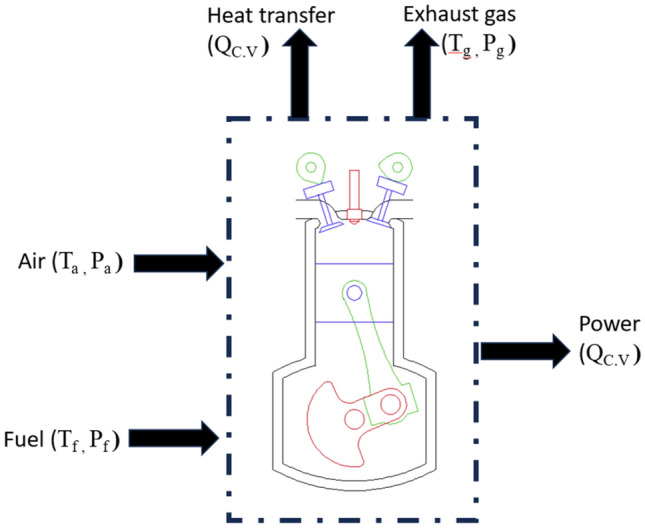
Engine control volume.

The schematic architecture shown in Fig. 3 serves as a visual representation of the theoretical thermodynamic model. The process of transferring input and output energy to and from the engine may be effectively understood by conceptualizing the engine as a control volume. The engine acquires energy in the form of chemical energy from the fuel, as well as the energy of the incoming air. The energy indicated before is transformed into output power, energy present in the exhaust gas, and heat transfer^24^. The study of the energy and exergy of the engine control volume shown in Fig. 3 assumes that the whole engine system functions under steady-state circumstances. It further presumes that the inlet air and gas from the exhaust may be treated as integrates of ideal gases, and any potential and kinetic energy variations are considered negligible or disregarded^25^.

### Energy analysis

To perform the energy balance, it is imperative to make the assumption that the engine is operating in a steady-state condition. The governing equation for energy analysis is presented below. The concept of energy balance is defined and explained in Eq. (1). Equation (2) can be derived from Eq. (1) through rearrangement. By substituting the value of Eqs. (3), (4) and (5) and ${\dot{Q}}_{air}$, the amount of energy loss can be determined. The energy efficiency is determined by Eq. (6)^25^.

1 $$\sum {En}_{in}= \sum {En}_{out},$$

2 $${\dot{Q}}_{air}+ {En}_{fuel}= \dot{W}+{En}_{exh}+{En}_{loss},$$

3 $${En}_{fuel}={\dot{m}}_{fuel}{CV}_{fuel},$$

4 $$\dot{W}= \frac{2\pi LN}{60},$$

5 $${En}_{exh}=\left({\dot{m}}_{fuel}+{\dot{m}}_{air}\right){C}_{p}{T}_{out},$$

6 $${\eta }_{En}=\frac{\dot{W}}{{\dot{m}}_{fuel}{CV}_{fuel}+{\dot{Q}}_{air}}.$$

Where ${\dot{Q}}_{air}$ is mass flow rate air, ${\dot{m}}_{fuel}$ is mass flow rate of fuel, where L and N are the load and speed of rotation of the engine, ${CV}_{fuel}$ is Calorific value of fuel, ${C}_{p}$ is specific heat of the exhaust gases and ${T}_{out}$ is the temperature at the engine outlet.

### Exergy analysis

The second law of thermodynamics finds its application, in analysing the exergy of a system. In this context “it” refers to the work that a system can achieve through interactions, with its environment. It can also denote the minimum effort required to reach a state considering the prevailing conditions. The concept of “energy quality” involves assessing the usefulness or value of energy quantitatively speaking. To achieve this objective, it is crucial to establish conditions known as the reference state. For this investigation we determined that the reference state has temperature and pressure values of T0 = 288.15 K and P0 = 101 kPa respectively^6^.

The principle of exergy conservation does not hold true, as exergy is actually subject to destruction. Hence, it is necessary to incorporate the term ‘exergy destruction’ into the exergy balance equation of every thermal system. The exergy balance equation for a steady-state open system is represented by Eq. (7)^6^.

7 $$\sum {Ex}_{in}= \sum {Ex}_{out}+\sum {Ex}_{dest},$$

8 $${Ex}_{air}+ {Ex}_{fuel}= {Ex}_{W}+{Ex}_{exh}+{Ex}_{loss}+{Ex}_{dest},$$

The Ex rate of the air reaching the experimental engine is determined through the utilization of Eq. (9).

9 $${Ex}_{air}={\dot{m}}_{air}\{{C}_{P.air}[\left({T}_{air}-{T}_{0}\right)-{T}_{0}{\text{ln}}\left(\frac{{T}_{air}}{{T}_{0}}\right) ]+R{T}_{0}{\text{ln}}\left(\frac{{P}_{air}}{{P}_{0}}\right)\}.$$

The variables T, Cp, and P denote temperature, specific heat capacity, and pressure correspondingly.

10 $${Ex}_{fuel}={\dot{m}}_{fuel}{\epsilon }_{fuel}LHV.$$

The variable “fuel ε” denotes the chemical exergy factor of fuel. The Eq. (11) is employed for the purpose of calculating the chemical exergy factor of a given fuel.

11 $${\epsilon }_{fuel}=1.0401+0.1728\frac{H}{C}+0.0432\frac{O}{C}+0.1728\frac{S}{C}\left[1-2.0628\frac{H}{C}\right].$$

The fuel’s mass fractions of hydrogen, carbon, oxygen, and sulphur are represented by the symbols H, C, O, and S, respectively.

12 $${Ex}_{W}=\omega T,$$

13 $${Ex}_{exh}=\sum_{i=1}^{n}{\dot{m}}_{i}\left({ex}_{tm,i}+{ex}_{ch,i}\right),$$

14 $${ex}_{tm,i}={C}_{P,i}\left[\left({T}_{exh}-{T}_{0}\right)-{T}_{0}{\text{ln}}\left(\frac{{T}_{exh}}{{T}_{0}}\right) \right]+{R}_{i}{T}_{0}{\text{ln}}\left(\frac{{P}_{exh}}{{P}_{0}}\right),$$

15 $${ex}_{ch,i}=\overline{R}{T }_{0}{\text{ln}}\left(\frac{{Y}_{i}}{{Y}_{env,i}}\right).$$

The universal gas constant, $\overline{R }$, is used to represent a constant value. The molar fraction of the ith compound in the exhaust gas is denoted as yi. Similarly, the molar fraction of the ith compound in the environment is represented as ${Y}_{env,i}$^6^.

16 $${Ex}_{loss}={\dot{Q}}_{loss}\left(1-\frac{{T}_{o}}{{T}_{engine}}\right).$$

By substituting the calculated values of Exair, Exfuel, ExW, Exexh, and Exloss into Eq. (17), it is possible to determine the exergy destruction rate of the experimental engine. Hence, the exergy efficiency of the experimental engine can be formulated as follows.

17 $${\eta }_{Ex}=\frac{{Ex}_{W}}{{Ex}_{fuel}+{Ex}_{air}}.$$

### Sustainability analysis

Sustainability is sometimes characterised as the act of preserving resources in order to satisfy the demands of future generations while concurrently addressing the needs of the present generation. The approach is based on the fundamental concepts of fostering economic progress, safeguarding the environment, and promoting social development. Clearly, these notions possess the capacity to achieve better efficiency in order to optimise the consumption of energy resources. As a result of this line of reasoning, the concept of exergy the significance of analysis in the field of sustainability analysis are noteworthy. There are approaches that are based on exergy. The purpose of this publication is to provide a comprehensive examination of the primary indicators used in the evaluation of process sustainability. The sustainability index is the reciprocal of the depletion number. The Eq. (18) of a sustainability index may be expressed in the following manner^6^.

18 $$SI=\frac{1}{DN}.$$

The depletion number, as described in Eq. (19), provides researchers with valuable information on the efficiency of the test fuel^14^.

19 $$DN=\frac{{Ex}_{dest}}{{Ex}_{in}}=\left(1-{\eta }_{Ex}\right).$$

### Progression of experiment

**Figure 4:**
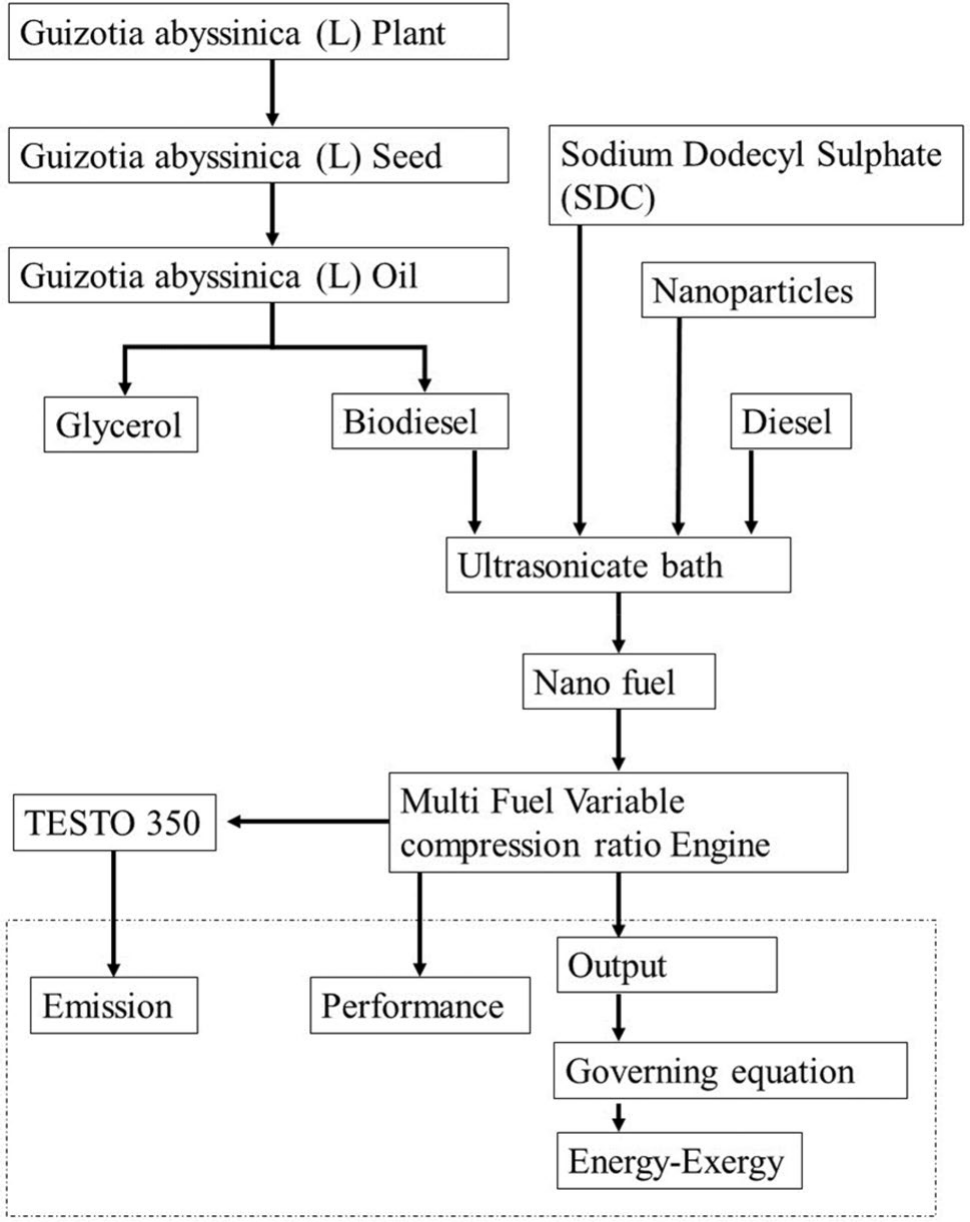
Step-wise flow diagram of experimental procedure.

The Fig. 4 denotes the comprehensive experimental technique used in this investigation, presented in a step-by-step manner with the aid of a flow diagram.

### Uncertainty analysis

A multitude of factors may have an impact on the degree of uncertainty within a particular scenario. The variables included in this study comprise many criteria, including the state and maintenance of the equipment, the operational status of the equipment, the environmental circumstances under which the equipment is used, and the precision of any collected data. The determination of uncertainty in this study is based on the square root approach, which is applied to the parameters being considered, as described by Eq. (20)^26^. The job in question is accompanied by a certain level of uncertainty, as indicated by the % value provided^27,28^. The prevailing degree of uncertainty is around 2.247%. Table 4 displays the level of uncertainty pertaining to the parameters of the inquiry.

**Table 4:** Uncertainty pertaining to the parameters.

Variables	Instrument	Unit	Uncertainty
Engine load	Load sensor (LS)	kg	± 0.5
Heating value	Heat value indicator (HVI)	MJ/kg	± 1.2
Load indicator	Analog meter (LI)	kg	± 0.2
Speed	Speed sensor (SS)	RPM	± 1.5
Specific fuel consumption	Fuel flow measuring unit (FFM)	–	± 0.05
Combustion chamber pressure	Pressure sensor (PS)	bar	± 0.1
Combustion chamber temperature	Temperature sensor (TS)	^0^ K	± 0.1
Testo 350 gas analyser			
Gas analyser	CO_2_	g/kWh	± 0.2
Gas analyser	NO_x_	ppm	± 1.0
Gas analyser	CO	g/kWh	± 0.1

20 $$U={\left[{\left(\frac{\partial R}{\partial {x}_{1}}{W}_{1}\right)}^{2}+{\left(\frac{\partial R}{\partial {x}_{1}}{W}_{2}\right)}^{2}+\dots +{\left(\frac{\partial R}{\partial {x}_{1}}{W}_{n}\right)}^{2}\right]}^{1/2},$$

21 $$U={\left[{\left({U}_{LS}\right)}^{2}+{\left({U}_{HVI}\right)}^{2}+{\left({U}_{LI}\right)}^{2}+{\left({U}_{SS}\right)}^{2}+{\left({U}_{FFM}\right)}^{2}+{\left({U}_{PS}\right)}^{2}+{\left({U}_{TS}\right)}^{2}+{\left({U}_{{NO}_{X}}\right)}^{2}+{\left({U}_{CO}\right)}^{2}+{\left({U}_{{CO}_{2}}\right)}^{2}\right]}^{1/2},$$

$$U={\left[{\left(0.5\right)}^{2}+{\left(1.2\right)}^{2}+{\left(0.2\right)}^{2}+{\left(1.5\right)}^{2}+{\left(0.05\right)}^{2}+{\left(0.1\right)}^{2}+{\left(0.1\right)}^{2}+{\left(0.2\right)}^{2}+{\left(1.0\right)}^{2}+{\left(0.1\right)}^{2}\right]}^{1/2},$$

$$U=\mp 2.247\%.$$

### Informed consent

This article is about consent to solar energy and agricultural research procedures ethics.

### Experiments and field studies on plants

The Study complies with local and national guidelines and regulations.

## Result and discussion

### Engine power output

**Figure 5:**
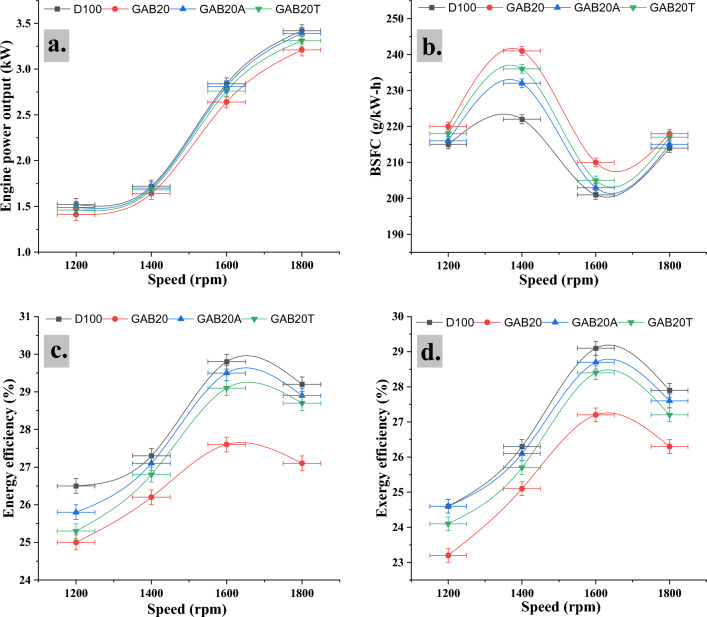
(**a**) Engine power output vs Engine speed, (**b**) BSFC vs Engine speed, (**c**) Energy efficiency vs Engine speed, (**d**) Exergy efficiency vs Engine speed.

Figure 5a demonstrates that at 1200 rpm the engine power output was 1.52, 1.41, 1.49 and 1.46 kW for D100, GAB20, GAB20A, and GAB20T respectively. For 1400 rpm the engine power output was 1.72, 1.64, 1.7 and 1.68 kW for D100, GAB20, GAB20A, and GAB20T respectively. For 1600 rpm the engine power output was 2.84, 2.64, 2.81 and 2.76 kW for D100, GAB20, GAB20A, and GAB20T respectively. For 1800 rpm the engine power output was 3.42, 3.21, 3.39 and 3.31 kW for D100, GAB20, GAB20A, and GAB20T respectively. The main variables that contribute to the observed occurrence are the reduction in mechanical friction loss and the improvement in volumetric efficiency of the engine at certain speeds. This finding is supported by past academic research^29^. It is found that maximum power output was noted for GAB20A at 1800 rpm out of all blend fuels. The results indicate that the use of binary blends consisting of biodiesel with Nano additive mixes lead to an improvement in engine power output in comparison to a blend composed only of biodiesel and diesel. The improvements in power output may be ascribed to modifications in fuel specification factors, such as viscosity, cetane number, and oxygen content^30^.

### Brake specific fuel consumption

The parameter often known as braking specific fuel consumption (BSFC) is used to measure the fuel consumption in proportion to the effective braking power. The brake specific fuel consumption (BSFC) has an inverse correlation with speed and a positive correlation with the proportion of biodiesel fuel^2^. The combustion effect in the engine cylinder is enhanced by the presence of nanoparticles in biodiesel, leading to an increase in the fuel’s momentum and propagation^31^. Figure 5b demonstrates that at 1200 rpm the BSFC was 215, 220, 216, and 218 g/kWh for D100, GAB20, GAB20A, and GAB20T respectively. For 1400 rpm the BSFC was 222, 241, 232, and 236 g/kWh for D100, GAB20, GAB20A, and GAB20T respectively. For 1600 rpm the BSFC was 201, 210, 203 and 205 g/kWh for D100, GAB20, GAB20A, and GAB20T respectively. For 1800 rpm the BSFC was 214, 218, 215, and 217 g/kWh for D100, GAB20, GAB20A, and GAB20T respectively. It is found that minimum fuel consumption was noted for GAB20A at 1600 rpm out of all blend fuels. The biodiesel has shown elevated BSFC values under various Speed levels. The GAB20A has displayed an improvement in BSFC, as seen in figure. The rise in fuel consumption may be linked to the decrease in the calorific value of the mixed fuel^14^.

### Energy efficiency

Figure 5c illustrates the variations in this parameter in relation to engine speed and energy efficiency for the 4 fuel mixtures investigated in this study. It can be observed that the proportion of energy efficiency demonstrates a parabolic pattern when considering different fuel types. This phenomenon suggests the presence of a positive correlation between engine speed and the observed trend^14^. However, once the engine speed surpasses a threshold ranging from 1600 rpm, a noticeable decrease becomes evident. The observed tendency can be primarily attributed to two factors: increased brake specific fuel consumption and exhaust gas loss of the engine at higher speeds. This conclusion is supported by previous studies conducted by other researchers^17,18^. Figure 5c demonstrates that at 1200 rpm the energy efficiency was 26.5, 25, 25.8, and 25.3% for D100, GAB20, GAB20A, and GAB20T respectively. For 1400 rpm the energy efficiency was 27.3, 26.2, 27.1, and 26.8% for D100, GAB20, GAB20A, and GAB20T respectively. For 1600 rpm the energy efficiency was 29.8, 27.6, 29.5, and 29.1% for D100, GAB20, GAB20A, and GAB20T respectively. For 1800 rpm the energy efficiency was 29.2, 27.1, 28.9, and 28.7% for D100, GAB20, GAB20A, and GAB20T respectively. It is found that maximum energy efficiency was noted for GAB20A at 1600 rpm out of all blend fuels. At the same time the minimum energy efficiency was noted for GAB20 at 1200 rpm out of all blend fuels. The thermal efficiency of a heat engine pertains to the percentage of heat that is efficiently converted into mechanical work during the burning of fuel^32^. Moreover, an enhanced thermal efficiency results in a reduced specific fuel consumption and lower fuel requirements for a given power output. Hence, the use of Nano additives in combination with biodiesel, particularly in biodiesel blends, enables the attainment of improved power or energy generation with reduced fuel consumption in comparison to biodiesel alone^19^.

### Exergy efficiency

Figure 5d depicts the correlation between the exergy of output power and the rotational speed of the engine. The data collected from the study suggest a strong correlation between the exergy efficiency results and the energy efficiency results. The exergy efficiency exhibits an upward pattern when the rotational speed escalates from 1200 revolutions per minute (rpm) to 1600 rpm, subsequently followed by a significant decrease. This suggests that there is a constant pattern in the trends of energy and exergy efficiency across all fuel combinations, although with varied magnitudes. It is worth noting that exergy efficiency regularly demonstrates lower magnitudes in comparison to energy efficiency. The results indicate a significant degree of agreement with the conclusions reported by prior researchers^30^. Figure 5d demonstrates that at 1200 rpm the exergy efficiency was 24.6, 23.2, 24.6, and 24.1% for D100, GAB20, GAB20A, and GAB20T respectively. For 1400 rpm the exergy efficiency was 26.3, 25.1, 26.1, and 25.7% for D100, GAB20, GAB20A, and GAB20T respectively. For 1600 rpm the exergy efficiency was 29.1, 27.2, 28.7, and 28.4% for D100, GAB20, GAB20A, and GAB20T respectively. For 1800 rpm the exergy efficiency was 27.9, 26.3, 27.6, and 27.2% for D100, GAB20, GAB20A, and GAB20T respectively. It is found that maximum exergy efficiency was noted for GAB20A at 1600 rpm out of all blend fuels. At the same time the minimum energy efficiency was noted for GAB20 at 1200 rpm out of all blend fuels. The use of biodiesel blends and other additives was shown to enhance the exergy efficiency of a compression ignition (CI) engine^33^.

### Energy-exergy analysis

**Figure 6:**
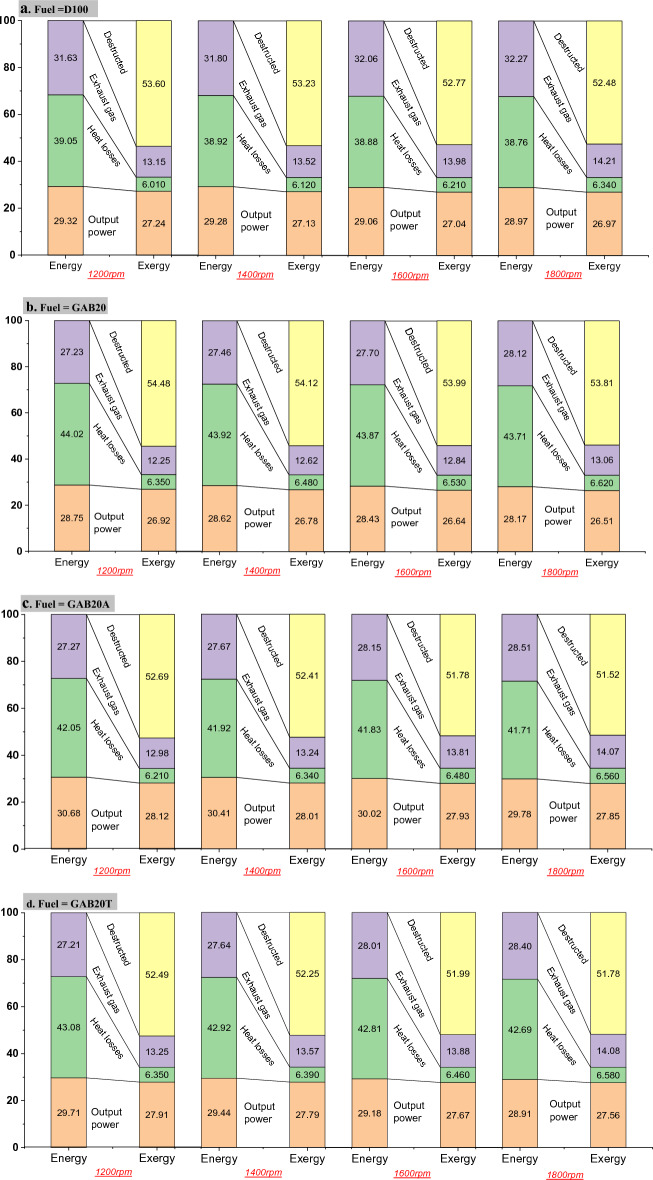
Energy exergy analysis (**a**) D100, (**b**) GAB20, (**c**) GAB20A and (**d**) GAB20T.

Figure 6a–d depicts the energy and exergy distribution of multiple fuels at engine speeds of 1200–1800 revolutions per minute (rpm). A comparative analysis was conducted to assess the exergy and energy of output power for 4 different fuels. The findings indicate that the exergy efficiency, represented as the percentage of output power exergy, is slightly lower compared to the percentage of output power energy. The evaluation of emission standards and engine performance heavily relies on the critical variables of the engine’s maximum torque and power occurring at certain speeds. The diagram illustrates the levels of output power energy and exergy, where the blue bars are used as visual aids. A comparative examination of the exergy and energy of output power for the four distinct fuels indicates that the exergy efficiency, represented as the proportion of output power exergy, exhibits a somewhat lower value compared to the proportion of output power energy. The average the output power energy was noted that 29.157, 28.492, 30.222, and 29.31% of total energy for D100, GAB20, GAB20A, and GAB20T fuel respectively. Where the average output power exergy was found to be 27.095, 26.712, 27.977, and 27.732% of total exergy for D100, GAB20, GAB20A, and GAB20T fuel respectively. The chemical exergy of each intake fuel type, specifically EFI, consistently exceeds the lower heating value (LHV). As a result, the exergy of the fuel would exceed its energy content, resulting in a reduction in exergy efficiency^34^.

Therefore, the significance of fuel quality or its availability consistently surpasses its heating value. The aforementioned finding has been substantiated by prior research inquiries carried out on various types of fuels and in different operational scenarios. The average the heat loss in energy analysis was noted that 38.902, 43.880, 41.877, and 42.875% of total energy for D100, GAB20, GAB20A, and GAB20T fuel respectively. Where the average heat loss in energy analysis was found to be 6.170, 6.495, 6.397, and 6.445% of total exergy for D100, GAB20, GAB20A, and GAB20T fuel respectively. Heat transfer losses are identified as the main factor contributing to energy inefficiency across various fuel combinations. However, in the context of exergy distribution analysis, it is seen that this specific component displays the least significant fraction. Hence, it can be deduced that the exergy related to heat transfer exhibits a much smaller magnitude compared to the energy connected with heat transmission, thereby suggesting that a substantial percentage of energy expended via heat transfer lacks the ability to generate any useful output. Upon comparing the fraction of energy dissipated as heat loss at the different speeds, it is evident that although the quantity of heat loss was marginally lower at the speed corresponding to maximum power, the proportion of heat loss exergy remained relatively constant at all speeds. The average exhaust gas energy analysis was noted that 31.94, 27.62, 27.9, and 27.81% of total energy for D100, GAB20, GAB20A, and GAB20T fuel respectively. Where the average exhaust gas exergy analysis was found to be 13.715, 12.692, 13.525, and 13.695% of total exergy for D100, GAB20, GAB20A, and GAB20T fuel respectively. The exergy loss via exhaust gases is more than that of heat transfer due to the fact that exergy is characterised as the fuel's quality or its availability. The average destructed exergy was found to be 53.02, 54.1, 52.1, and 52.127% of total exergy for D100, GAB20, GAB20A, and GAB20T fuel respectively. The finding indicates that a significant portion of the exergy distribution for the 4 fuel combinations, evaluated at the 4 different speeds, was either lost owing to irreversibility or led to the degradation of exergy. The exergy destructed constituted at least 50% of the whole amount. The majority of exergy supplied to the engine is seen to be squandered due to irreversibility. In contrast, the first law analysis and energy balance merely account for losses in terms of heat transfer and exhaust gas energy. This phenomenon has the potential to result in a diminished exergy efficiency when compared to energy efficiency. The acquired performance values demonstrate a significant concurrence with those reported in the extant literature^24^.

### Sustainability analysis

**Figure 7:**
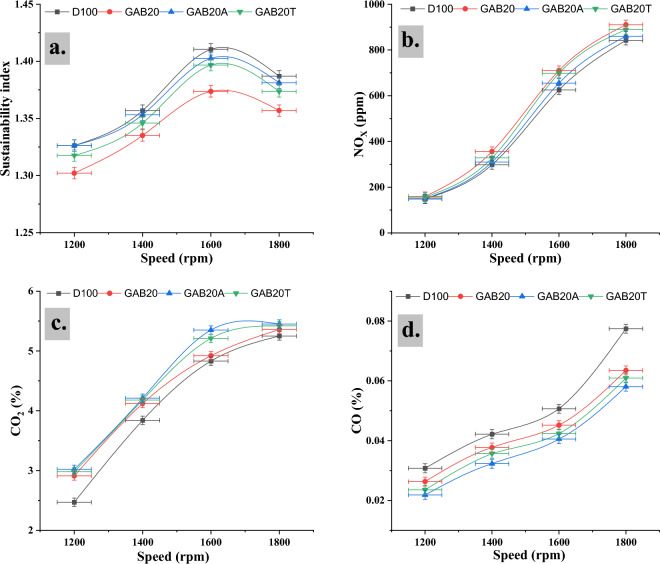
(**a**) Sustainability index vs engine speed, (**b**) Nitrogen oxides vs engine speed, (**c**) Carbon dioxide vs engine speed, (**d**) Carbon monoxide vs engine speed.

A sustainability analysis evaluates the environmental, social, and economic impact of a given practice, product, or policy over its entire life cycle. The findings shown Fig. 7a illustrate that the test engine’s capacity for improvement displayed a gradual rise with each successive increase in engine speed, irrespective of the fuel variant used. The GAB20A and GAB20T materials demonstrated the most significant and least significant possibilities for improvement, respectively, for every level of engine speed. For 1200 rpm the sustainability index was 1.326, 1.302, 1.326, and 1.317 for D100, GAB20, GAB20A, and GAB20T respectively. For 1400 rpm the sustainability index was 1.356, 1.335, 1.353, and 1.345 for D100, GAB20, GAB20A, and GAB20T respectively. For 1600 rpm the sustainability index was 1.410, 1.373, 1.402, and 1.396 for D100, GAB20, GAB20A, and GAB20T respectively. For 1800 rpm the sustainability index was 1.386, 1.356, 1.381, and 1.373 for D100, GAB20, GAB20A, and GAB20T respectively. It is found in Fig. 6 that maximum sustainability index was noted for GAB20A at 1600 rpm out of all blend fuels. The enhanced potential for enhancement in the test engine for biodiesel may be attributed to its relatively lower exergy efficiencies and higher rates of exergy degradation^30^. The test engine’s depletion number shown a marginal decline when the engine load was augmented for every fuel variant. The recorded depletion values for each load exhibited the largest magnitude for GAB, followed by D100, GAB20, GAB20A, and GAB20T in sequential order. The depletion values of the test engine were measured to be 0.713 for GAB20A, value as under a speed of 1600 rpm.

### Nitrogen dioxide

There have been reports suggesting a potential correlation between the utilisation of biodiesel fuel in combination with advanced injection timing and the observed increase in NOx emissions^35^. Figure 7b demonstrates that at 1200 rpm the NO_x_ emission was 148, 159, 150, and 156 ppm for D100, GAB20, GAB20A, and GAB20T respectively. For 1400 rpm the NO_x_ emission was 298, 356, 310, and 328 ppm for D100, GAB20, GAB20A, and GAB20T respectively. For 1600 rpm the NOx emission was 625, 710, 655, and 698 ppm for D100, GAB20, GAB20A, and GAB20T respectively. For 1800 rpm the NOx emission was 842, 910, 860 and 889 ppm for D100, GAB20, GAB20A, and GAB20T respectively. It is found that minimum NO_x_ emission was noted for GAB20A at 1200 rpm out of all blend fuels. The utilisation of unsaturated biodiesels has the potential to increase the adiabatic flame temperature, which in turn may lead to elevated levels of NO_x_ emissions. The combustion process occurs without any ignition delay, it may lead to elevated cylinder temperatures, which in turn can cause an escalation in NOx emissions. As the demand on the test engine grew, there was a corresponding increase in the emissions of nitrogen oxides^30^.

### CO_2_ emission

Furthermore, there have been reports indicating a positive correlation between enhanced combustion efficiency and elevated levels of carbon dioxide (CO_2_) emissions^36^. When a fuel exhibits a high density, it signifies that a larger quantity of mass is being fed into the combustion chamber within a certain volume. The rise in CO_2_ emission levels stemming from the use of biodiesel may be ascribed to the following factor^37^. Figure 7c demonstrates that at 1200 rpm the CO_2_ emission levels was 2.47, 2.91, 3.02, and 2.98% for D100, GAB20, GAB20A, and GAB20T respectively. For 1400 rpm the CO2 emission levels was 3.84, 4.12, 4.21, and 4.18% for D100, GAB20, GAB20A, and GAB20T respectively. For 1600 rpm the CO_2_ emission levels was 4.83, 4.92, 5.35, and 5.21% for D100, GAB20, GAB20A, and GAB20T respectively. For 1800 rpm the CO_2_ emission levels was 5.25, 5.36, 5.45, and 5.42% for D100, GAB20, GAB20A, and GAB20T respectively. It is found that maximum CO_2_ emission levels was noted for GAB20A at 1800 rpm out of all blend fuels. The introduction of a larger amount of gasoline into the engine cylinder is associated with an observable rise in emissions^38^.

### CO emission

The air-to-fuel ratio plays a crucial role in the production of CO emissions. Insufficient air introduced into the fuel–air mixture leads to incomplete combustion and the presence of unburned fuel^19^. In addition, an important contributing factor to the production of CO is the insufficient blending of air and fuel. The presence of this deficiency results in the creation of fuel-rich regions, characterised by a limited supply of oxygen that hinders the conversion of CO to carbon dioxide (CO_2_)^39^. Figure 7c demonstrates that at 1200 rpm the CO emissions was 0.0308, 0.0263, 0.0219, and 0.0236% for D100, GAB20, GAB20A, and GAB20T respectively. For 1400 rpm the CO emissions was 0.0422, 0.0377, 0.0323, and 0.0357% for D100, GAB20, GAB20A, and GAB20T respectively. For 1600 rpm the CO emissions was 0.0507, 0.0452, 0.0406, and 0.0424% for D100, GAB20, GAB20A, and GAB20T respectively. For 1800 rpm the CO emissions was 0.0775, 0.0635, 0.0581, and 0.0609% for D100, GAB20, GAB20A, and GAB20T respectively. It is found that minimum CO emissions was noted for GAB20A at 1200 rpm out of all blend fuels. The assertion is substantiated by the observation of increased oxygen concentration in carbon chains of shorter length, resulting in improved efficiency and thoroughness of the combustion process^40^. Furthermore, it is important to mention that methyl esters possessing longer carbon chain lengths exhibit increased boiling and melting temperatures. As a result, these esters are less prone to complete vaporisation and combustion. As a result, this particular attribute leads to a higher emission of CO^10^.

### Validation and justification

**Figure 8:**
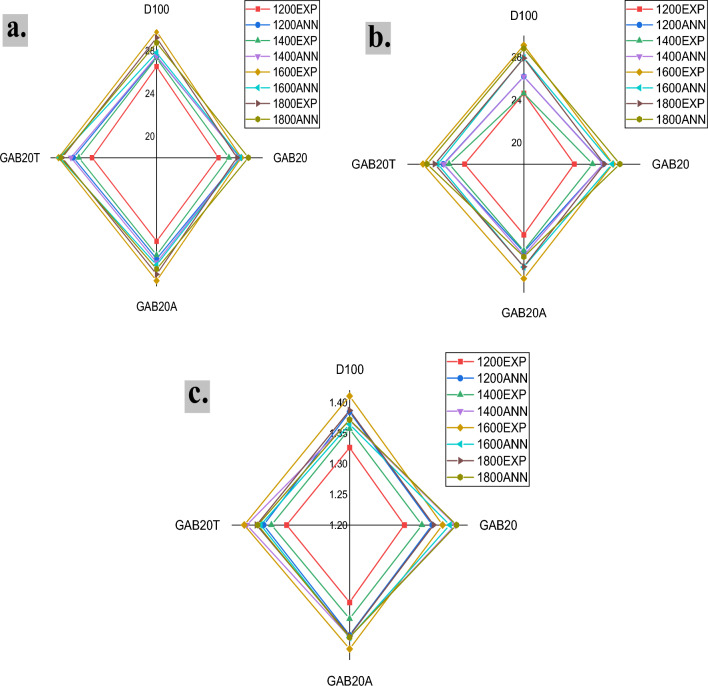
**(a**) Validation of energy % by ANN, (**b**) validation of exergy % by ANN, (**c**) validation of sustainability index by ANN.

The other characteristics, such as engine power, brake specific fuel consumption (BSFC), and emission parameters, are denoted by numerical values accompanied by their corresponding units. The validation procedure takes into account dimensionless quantities such as energy efficiency, exergy efficiency, and sustainability index. The value was estimated via the artificial neural network (ANN) computational method^28,41^. The obtained experimental value is later compared to the outputs generated by the ANN, as shown in Fig. 8a–c. The Eq. (22) is used to compute the percentage difference between the outputs of an ANN and the equivalent experimental results. The mean discrepancy was computed for the corresponding variables, yielding disparities of 1.703%, 2.246%, and 1.416% for energy efficiency, exergy efficiency, and sustainability index, respectively.

22 $$Percentage \,diffrence=\left[\left(ANN-EXP\right)/\left(\frac{ANN+EXP}{2}\right)\right]\times 100.$$

### Comparative analysis

There exists a multitude of sources for biodiesel, as seen from the available evidence. In the current investigation, the *Guizotia* *abyssinica* (L.) oil selected for the experimental analysis. After obtaining the trial result, a comparison analysis is conducted by juxtaposing it with the other biodiesel mix. Through this analysis, we can ascertain the position of the biodiesel used in the research. The energy efficiency level exhibits a decrease of 7.2% and exergy efficiency level exhibits a decrease of 4.38% compared to the average value observed in all respective previous studies^14,17,18,30^ (waste cooking oil, jatropha, botryococcus braunii, palm and opium poppy oil) taken into account. The sustainability index level exhibits a decrease of 6.098% compared to the average value observed in all previous studies^14,17,18,30^ (waste cooking oil, jatropha, botryococcus braunii, palm, and opium poppy) taken into account. Further the previous research comparison with the present work is shown in the Table 5.

**Table 5:** Previous literatures compared with present work.

Engine specification	Biodiesel/alcohol	NP/size	NPs dosage	BSFC	${\eta }_{En}$	${\eta }_{Ex}$	SI	NO_x_	CO_2_	CO	Ref
Single-cylinder, four-stroke, naturally aspirated, water cooled CI engine. CR = 18	Jatropha, Ethanol			↓	↑	↑	↑	↑		↑	^14^
Four-stroke, four-cylinder, naturally aspirated, water cooled, DI engine. CR = 17.8	Butanol	Aluminum oxide	30, 50 and 100 mg/l	↑				↓	↓	↑	^19^
Single-cylinder, naturally aspirated, water-cooled, a DI diesel engine. CR = 17.5, 3300 max rpm, Injection pressure 190 bar	Waste cooking oil, butanol				↓	↓	↑				^18^
Kirloskar Four stroke, single cylinder DI diesel engine, air cooled. 1500 rpm, 5.2 kW	Garcinia oil	Cerium oxid, Zirconium oxide and Titanium dioxide	25 ppm	↑				↓	↓	↓	^42^
Four-stroke, four-cylinder, naturally aspirated, direct-injected diesel engine. Steady load	Opium poppy and palm oils				↑	↑	↓				^17^
Fiat direct-injection, four-cylinders diesel engine with water cooled		Aluminum oxide and zinc oxide	50 and 100 ppm	↓				↑		↑	^40^
Single-cylinder, four-stroke, Direct-injection diesel engine. CR = 17.5, 1500 rpm, Injection pressure 220 bar	Botryococcus braunii microalgae biodiesel	Cerium Oxide and Copper Peroxide	100 ppm	↑	↑	↑	↓	↑			^30^

### Limitations

All research studies likely have limitations in technique, study design, materials, and other areas. The following restrictions may affect study results. The study publication must acknowledge and address any restrictions to tell readers about any limits that may impact the research results. Current study contains limitations like prior academic studies.

- Limited outcome parameters explored due to instrument constraints.
- In present study engine speed (rpm) was varied at full load condition. The variable load condition and variable compression ratio condition may result in different result.
- The present study emphasises only Al_2_O_3_ and TiO_2_ nanoparticles so the outcome not investigated for other Nano particles.

## Conclusions

This experimental study examines impact of aluminium oxide (Al_2_O_3_) and titanium dioxide (TiO_2_) nanoparticles as additions in biodiesel fuel derived from *Guizotia abyssinica* (L.) oil, focusing on their effects on performance and emissions. The assessment of fuel blends, which were created by combining nanoparticles and biodiesel, was conducted using energy, exergy, and sustainability indices. From 1200 to 1800 rpm, with 200 rpm jumps.

- Through research it found that sodium dodecyl sulphate (SDS) (CH_3_ (CH_2_)11SO_4_ − Na +) surfactants in a weight ratio of 1:2 with Nano particle was used showed better homogeneity of nanoparticles.
- The investigation reveals that the highest power production of 3.39 kW was seen for gasoline GAB20A at an engine speed of 1800 rpm, surpassing all other blended fuels.
- The analysis aids that minimum fuel consumption of 203 g/kWh was noted for fuels GAB20A at 1600 rpm out of all blend fuels.
- The research findings indicate that the GAB20A fuel blend had the highest energy efficiency of 29.5% and exergy efficiency of 28.7% when operated at 1600 rpm, surpassing all other fuel blends examined in the study.
- The study revealed that the GAB20A blend fuel exhibited the lowest NOx emission level of 150 ppm and CO emissions of 0.0219% when tested at an engine speed of 1200 rpm, compared to other blend fuels.
- The mean discrepancy was computed for the corresponding variables, yielding disparities of experimental values with ANN outputs was 1.703%, 2.246%, and 1.416% for energy efficiency, exergy efficiency, and sustainability index, respectively.
- It is evident that the NO_X_ emission exhibits a decrease of 21.34% and CO emission reduced more than 3/4 compared to the average value observed in all respective previous studies reported.

### Scope for future work

The study limitations or constraints include several domains that will provide guidance for future investigations and assist researchers in devising strategies and executing experimental endeavours within the realm of diesel engines. The following sections delineate prospective pathways for future researchers to investigate:

- Present study involves *Guizotia abyssinica* (L.) biodiesel and Al_2_O_3_ and TiO_2_ nanoparticles combination. There are several other biodiesel sources as well as different nanoparticles their combination can be explored.
- In present study sodium dodecyl sulphate (SDS) (CH_3_(CH2)11SO_4_ − Na +) surfactants in a weight ratio of 1:2 was used weight ratio with Nano particle can be varied and explored for better homogeneity.
- More output parameters can be explored of IC engine.
- Different numerical tool can be used for analysing the experimentally obtained results.

## Data Availability

The datasets used and/or analysed during the current study are available from the corresponding author on reasonable request.

## Abbreviations

GA: *Guizotia abyssinica *(L.)
GAB: *Guizotia* *abyssinica* biodiesel
Al_2_O_3_: Aluminium oxide
TiO_2_: Titanium dioxide
GAB20: Diesel 80% + Biodiesel 20%
GAB20T: Diesel 80% + Biodiesel 20% + TiO_2_ 100 ppm
GAB20A: Diesel 80% + Biodiesel 20% + Al_2_O_3_ 100 ppm
En: Energy
Ex: Exergy
SI: Sustainability index
BSFC: Break specific fuel consumption
NO_x_: Nitrogen oxide
CO_2_: Carbon dioxide
CO: Carbon monoxide
